# Devastating Worsening Renal Function Following the Initiation of Sacubitril/Valsartan: Falling Between Two Stools

**DOI:** 10.7759/cureus.46757

**Published:** 2023-10-09

**Authors:** Akinori Sairaku, Yukiko Nakano

**Affiliations:** 1 Cardiology, National Hospital Organization Higashihiroshima Medical Center, Higashihiroshima, JPN; 2 Cardiovascular Medicine, Hiroshima University Graduate School of Biomedical and Health Sciences, Hiroshima, JPN

**Keywords:** hypertension, worsening kidney function, sacubitril/valsartan, elderly, decompensated heart failure

## Abstract

Previously prescribed antihypertensive agents were switched to the maximum dose of sacubitril/valsartan, without any upward titration, in two elderly hypertensive patients with chronic kidney disease who were hospitalized for treatment of congestive heart failure, in the hope of its decongestive, antihypertensive, and potential renoprotective actions. However, they quickly fell into oliguria and finally required hemodialysis. Sacubitril/valsartan should be initiated with careful attention in elderly patients with advanced renal dysfunction.

## Introduction

Sacubitril/valsartan is one of the standard anti-heart failure drugs [1]. Given that it has a strong blood pressure-lowering effect as well [2], it has recently been approved for the treatment of essential hypertension in a few nations. Although its safety has been well-established, careful attention should be paid when it is introduced in elderly hypertensive patients with multiple comorbidities. We, in this report, describe two cases in which switching from previously prescribed antihypertensive agents to sacubitril/valsartan resulted in catastrophic worsening renal function (WRF).

## Case presentation

Case 1

**Figure 1:**
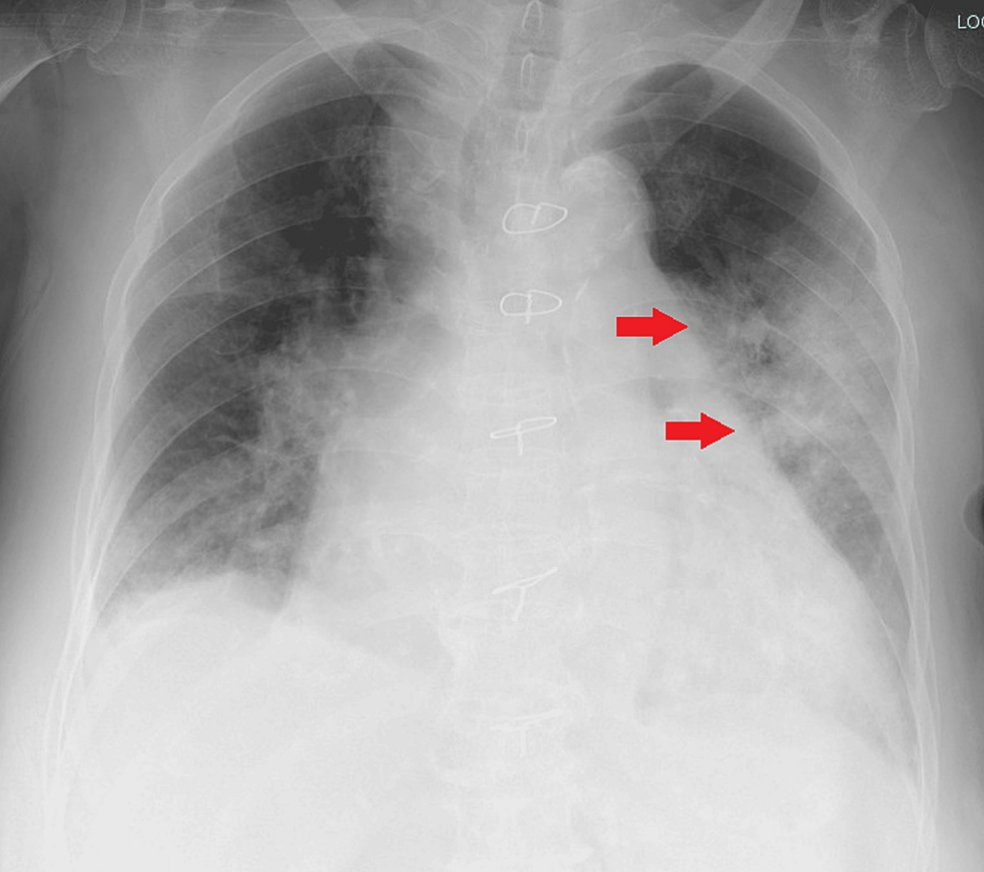
Chest X-ray on hospital admission in case 1. A localized infiltration (red arrows) was noted in the left upper lobe.

An 87-year-old woman was taken to our hospital for dyspnea. A chest X-ray showed a localized infiltration of the left upper lobe with an increased C-reactive protein level of 11.9 mg/dL (Figure 1).

**Figure 2:**
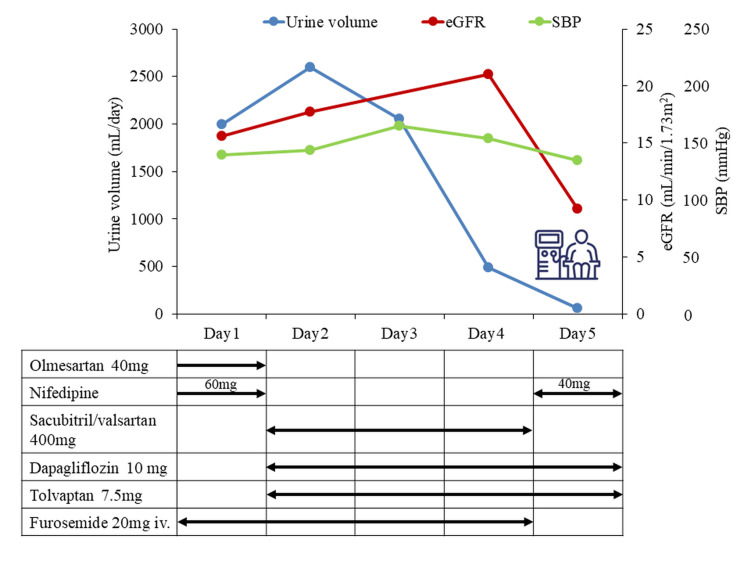
Clinical course and medical treatment given in case 1. SBP: systolic blood pressure, eGFR: estimated glomerular filtration rate An illustration of the medical machinery next to the seated person indicates hemodialysis.

We diagnosed her with pneumonia. She received an atrial septal defect closure operation at the age of 47 years old. Other comorbidities included hypertension, chronic kidney disease with an estimated glomerular filtration rate (eGFR) of 15.6 mL/min/1.73m^2^, and persistent atrial flutter. She was hospitalized, and oxygen administration and intravenous ceftriaxone 1000 mg/day were started. Although her left ventricular ejection fraction was within the normal limits, the tibial edema and an increased BNP level of 333.8 pg/mL suggested the coexistence of congestive heart failure. Based on that diagnosis, 20 mg of furosemide was infused once a day. A 7.5 mg of tolvaptan and 10 mg of dapagliflozin were also started on the day after admission. Previously prescribed antihypertensive agents including 40 mg of olmesartan and 60 mg of nifedipine were switched to 400 mg of sacubitril/valsartan in the hope of both its antihypertensive and anti-heart failure actions on day 2, as well. Her clinical condition appeared to improve on the second and third days. However, her urine volume abruptly dropped on day 4. She finally fell into anuria on day 5. Her eGFR sharply declined to 9.2 mL/min/1.73m^2^ after its transient improvement. Dyspnea quickly relapsed due to pulmonary congestion, and the use of noninvasive positive pressure ventilation was required. Hemodialysis was promptly introduced (Figure 2). All oral medications including sacubitril/valsartan were withdrawn. Her blood pressure was acceptably controlled, and no hyperkalemia was encountered prior to the initiation of dialysis during her hospital stay. Despite the careful and intensive treatment, her renal function never recovered. She was transferred to a sanatorium where regular hemodialysis was available on day 41.

Case 2

**Figure 3:**
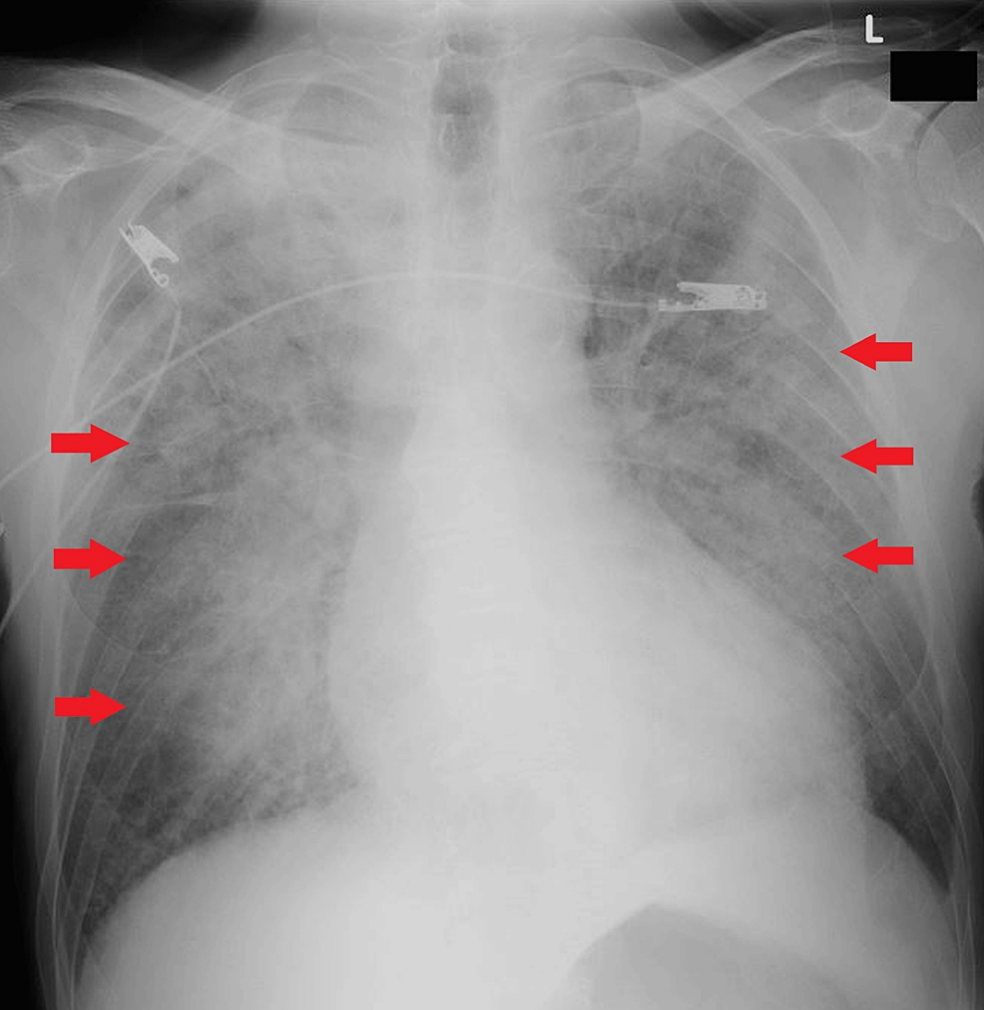
Chest X-ray on hospital admission in case 2. A typical butterfly shadow is noted (red arrows).

**Figure 4:**
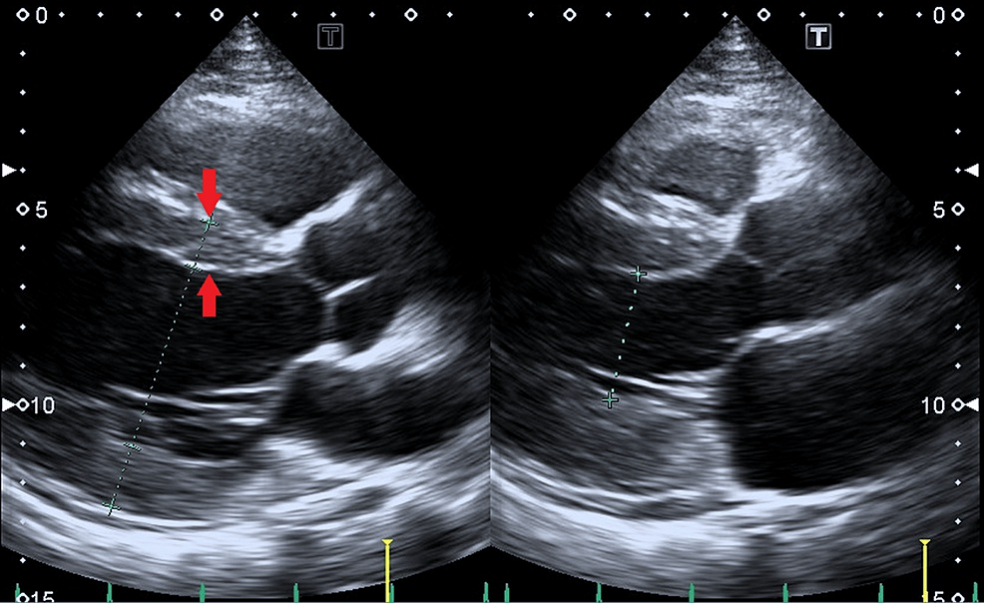
Echocardiogram on hospital admission in case 2. The left and right panels indicate diastolic and systolic phases of parasternal long-axis views, respectively. Preserved left ventricular ejection fraction of 59% and concentric left ventricular hypertrophy with interventricular septum thickness of 13 mm (red arrows) were noted.

An 83-year-old man was transferred from a clinic to our hospital for sudden onset orthopnea. A typical butterfly shadow on the chest X-ray and severe hypertension of 190/166 mm Hg suggested flash pulmonary edema due to acute decompensated heart failure (Figure 3). An echocardiogram showed a preserved left ventricular ejection fraction with left ventricular hypertrophy (Figure 4).

**Figure 5:**
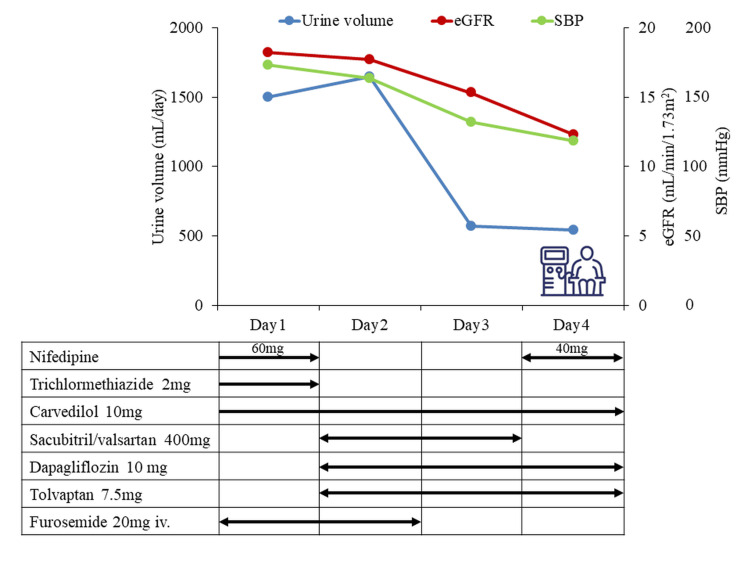
Clinical course and medical treatment given in case 2. SBP: systolic blood pressure, eGFR: estimated glomerular filtration rate

He had received an antihypertensive treatment with 60 mg of nifedipine, 2 mg of trichlormethiazide, and 10 mg of carvedilol for years. He was previously diagnosed with advanced chronic kidney disease. He was admitted to the intensive care unit. Noninvasive positive pressure ventilation, furosemide 20 mg injection, and an intravenous nitroglycerin drip were carried out without delay. A 7.5 mg of tolvaptan and 10 mg of dapagliflozin were started on day 2. In order to achieve better antihypertensive and decongestive effects, nifedipine and trichlormethiazide were replaced by 400 mg of sacubitril/valsartan on the day after admission (Figure 5). His dyspnea and blood oxygen level gradually ameliorated. His blood pressure control also improved progressively without any significant hypotension. However, he developed oliguria on days 3 and 4. His eGFR declined from 18.2 to 12.3 mL/min/1.73m^2^ without an increase in the potassium level. All oral medications including sacubitril/valsartan were stopped. Hemodialysis was introduced to ease the worsening dyspnea. It took 16 days for him to be weaned from the dialysis. His eGFR improved to 20.7 mL/min/1.73m^2^ on the day of hospital discharge (day 28).

## Discussion

A decrease in the renal blood flow resulting from a drop in the cardiac output and the administration of a loop diuretic and renal congestion elicited by fluid retention are well-known to contribute to the development of WRF in patients with acute decompensated heart failure [3]. Those factors were probably responsible for the catastrophic WRF incidences seen in the two cases to a greater or lesser extent. We however found that, in both cases, even the clinical course improved for a while after the initiation of the anti-heart failure treatment. In addition, that treatment resulted in neither a steep decline in blood pressure nor massive diuresis in either case. It would thus be reasonable to suspect another contributor rather than those stated above.

Both patients had already been taking potent antihypertensive agents with their maximum doses. Nevertheless, the blood pressure was not well-controlled in either of them upon their hospital admission. They thus needed not only an anti-heart failure treatment but also an intensification of the antihypertensive therapy. Also, considering their advanced renal insufficiency, some type of reno-friendly treatment was favorable. Taking those together, sacubitril/valsartan sounded like a kill-“three”-birds-with-one-stone treatment option because it has (1) an established decongestive action [1], (2) strong blood-pressure-lowering effect [1,2], and (3) potential renal protection effect [1,4].

Among sacubitril/valsartan-naïve patients with renal impairment, it is recommended that it should be started with the starting dose, and the dose should be gradually stepped up. We, however, were reluctant to follow that recommendation for the following concern. We were worried that the blood pressure control would be sacrificed during the early period of switching from the original antihypertensive agents to the starting dose of sacubitril/valsartan, resulting in further worsening of the heart failure in those cases. We thus decided to give them the maximum dose without any upward titration in order to avoid that potential risk.

It is well known that renin-angiotensin-aldosterone system (RAAS) inhibitors occasionally decrease renal function, especially during the early introduction period. This phenomenon is usually of no significance and even transient [5]. We inferred that an unusually serious RAAS inhibitor-induced WRF occurred after the initiation of sacubitril/valsartan in our second case. That inference may have validity because he was RAAS inhibitor-naïve and his WRF incidence was reversible. However, that was not the case in our first case because she had already been taking a strong angiotensin Ⅱ receptor blocker before the hospitalization. Therefore, it is uncertain whether the initiation of the sacubitril/valsartan had something to do with her renal death incidence. Yet, one thing for sure was that she never enjoyed the renal protection effect that we expected. Importantly, most previous trials did not test the efficacy and safety of sacubitril/valsartan in subjects with an eGFR of <30 mL/min/1.73m^2^ just like our cases [1,4]. Further, its pharmacological mechanism is complicated and has not been fully elucidated. For example, clinicians should keep in their mind its pharmacodynamic characteristics in subjects with renal impairment. Valsartan is primarily excreted via the biliary route, and thus its blood level in subjects with renal impairment is comparable to that of those with normal kidney function. On the contrary, sacubitril, which is a metabolite of sacubitril, is largely eliminated through the renal route. Therefore, renal impairment significantly increases its exposure [6]. Whether or not that has some clinical impact is not yet known, though.

It is common for clinical practice in aging societies to encounter elderly patients like our cases who have all of the following three conditions: hypertension, advanced renal dysfunction, and congestive heart failure. Given that sacubitril/valsartan has become increasingly popular, therefore, there would be a concern that the same mistake we had would be repeatedly made. We hope the present report will be taken as a wake-up call.

## Conclusions

We described two cases in which switching from previously prescribed antihypertensive agents to the maximum dose of sacubitril/valsartan, without any upward titration, resulted in devastating WRF. This was contrary to the renoprotective action we had expected. The introduction of sacubitril/valsartan should be approached with caution, especially in elderly patients with advanced renal impairment.
